# Unusual foreign body in the ear in an adult patient with psychiatric illness

**DOI:** 10.4103/0019-5545.49467

**Published:** 2009

**Authors:** Sanjay Arora, Sandeep Kumar Goyal

**Affiliations:** Department of ENT, MMIMSR, Mullana (Ambala), India. E-mail: drsanjayarora@sify.com; 1 Department of Psychiatry, MMIMSR, Mullana (Ambala), India

Sir,

Foreign body in ear is a common problem in pediatric population. In adults mostly we encounter accidental animate foreign body like cockroaches. Presence of inanimate foreign body in ear of an adult patient is very rare. We present a case of an adult patient with an unusual foreign body, beedi (a south Asian form of cigarette made by rolling tobacco in a tendu leaf) in his ear. We attribute this foreign body to psychiatric illness of the patient and propose to discuss that all adult patients with inanimate foreign body ear should be subjected to psychiatric evaluation.

Foreign bodies in the external auditory canal are a common presentation to the ENT department. The profile of foreign bodies in children's ears has been reported from a number of countries, however the adult experience is not well documented. Most reports of foreign bodies in the ears of adults have consisted of isolated, interesting cases. Unpublished data from the John Hopkins emergency department (US) 1987 found that the most common aetiology in adults (85% in 106 patients) was accidental entry of insects, 50% of these being cockroaches.[1] Bressler *et al*, (US) 1993 also found cockroaches to be the most common foreign body amongst 98 patients, although they did not differentiate between adult and paediatric groups.[2] Antonelli *et al*, (US) similarly report beads and insects, particularly cockroaches; to be the commonest foreign bodies in the external auditory canal from 273 combined paediatric and adult patients.[3] Ryan *et al*, found the majority of foreign bodies in adult patients to be the cotton wool tips of cotton buds. These are frequently used by the general population for cleaning or itching of the external ear canal.[4] We discuss a case of unusual foreign body in ear of an adult patient.

We present the case of a 32-year-old male patient, Mr. R who was taking treatment for schizophrenia. He was on maintainence therapy with Olanzapine 10mg before sleep with no active symptoms. He was on regular treatment in our psychiatric outpatient department since last 1 year.

**Figure 1:**
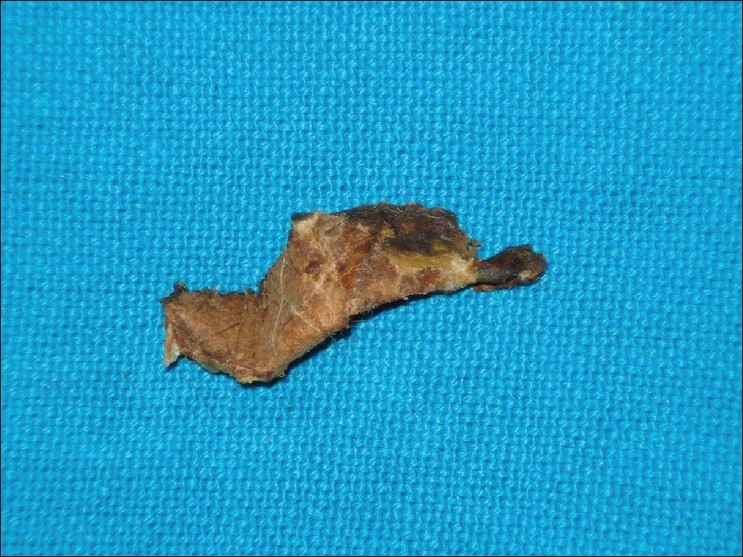
Removed foreign body (Beedi) from the patient's ear

Presently on a regular follow up visit he complained of blockage and decreased hearing in his left ear. He was promptly referred to the ENT department. On examination of the left ear a brownish mass was seen in the external auditory canal that was presumed to be wax. On removal (by Jobson Horne probe) the mass proved to be a beedi [Figure 1]. The patient even on persistent questioning did not remember how he had inserted a bidi in his ear.

After removal the ear canal was clean with a normal tympanic membrane. He was sent home with a prescription of prophylactic topical antibiotic eardrops and his routine psychiatric treatment.

Now, after 2 weeks the patient has no ear complaint.

Foreign body ear is a common occurrence but in paediatric population. Presence of foreign body in adult patient other than accidental entry of insects is not very common. Apart from this people insert inanimate things like cotton buds to relieve itching in their ears with full knowledge of having a foreign body in their ear. Presence of any inanimate foreign body in an adult patient without his knowledge is not well documented.

We propose that any adult patient who presents with complaints of ear blockage or decreased hearing and is diagnosed as having foreign body in his ear (without his or her knowledge) should be subjected to a thorough psychiatric evaluation.
